# Evaluation of Melanogenesis in A-375 Cells in the Presence of DMSO and Analysis of Pyrolytic Profile of Isolated Melanin

**DOI:** 10.1100/2012/854096

**Published:** 2012-05-02

**Authors:** Ewa Chodurek, Arkadiusz Orchel, Joanna Orchel, Sławomir Kurkiewicz, Natalia Gawlik, Zofia Dzierżewicz, Krystyna Stępień

**Affiliations:** ^1^Department of Biopharmacy, Medical University of Silesia, Narcyzów 1, 41-200 Sosnowiec, Poland; ^2^Department of Molecular Biology, Medical University of Silesia, Narcyzów 1, 41-200 Sosnowiec, Poland; ^3^Department of Instrumental Analysis, Medical University of Silesia, Narcyzów 1, 41-200 Sosnowiec, Poland; ^4^Department of Biotechnology and Genetic Engineering, Medical University of Silesia, Narcyzów 1, 41-200 Sosnowiec, Poland

## Abstract

The increase of a skin malignant melanoma (*melanoma malignum*) incidence in the world has been observed in recent years. The tumour, especially in advanced stadium with metastases, is highly resistant to conventional treatment. One of the strategies is to modulate melanogenesis using chemical compounds. In this study, the processes of differentiation and melanogenesis induced by dimethylsulfoxide (DMSO) in human melanoma cells (A-375) were investigated. Natural melanin isolated from A-375 melanoma cell line treated with 0.3% DMSO was analyzed by pyrolysis-gas chromatography-mass spectrometry (Py-GC/MS) method. The products derived from pheomelanin have not been stated in the pyrolytic profile of analyzed melanin. Within all products derived from eumelanins, 1,2-benzenediol has been predominated. It has been shown that in the melanoma cells stimulated with 0.3% and 1% DMSO, the increase of transcriptional activity of the tyrosinase gene took place. It was accompanied by the rise of tyrosinase activity and an accumulation of melanin in the cells. The better knowledge about the structure of melanins can contribute to establish the uniform criteria of malignant melanoma morbidity risk.

## 1. Introduction

In recent years, the increase of a skin malignant melanoma (*melanoma malignum*) incidence in the world has been observed. Though this type of carcinoma comprises just 5% of all malignant skin cancers, it is also responsible for 75–80% of deaths caused by these tumors. The highest morbidity rate of this cancer was noted in Australia (40–60/100 000), in the United States of America (10–20/100 000), and in Europe (10–15/100 000) [1, 2].

The morbidity rate of skin *melanoma malignum* in Poland, according to the National Registry of Cancer, amounted 4.4/100 000 and 5.1/100 000 in 2002, for men and women, respectively. It was observed that the amount of new cases of the melanoma in Poland from 1982 to 2002 increased 3 times and the rate of death in the same period increased over 2 times, what was attested to a better efficiency of therapy of this cancer in recent years [3].

The high morbidity rate is also associated with rapidly growing mortality rate. The malignant melanoma at higher stages of malignancy is highly resistant to conventional treatment. Therefore, there is a demand for the development of alternative antitumor therapies and better understanding of the mechanisms responsible for the malignant phenotypes [4].

*Melanoma malignum* is one of the most malignant and at the same time the most frequently occurring aggressive skin tumor, the origin of which is the malignant transformation of melanocytes, cells which produce the melanin pigment [4, 5]. Melanocytes of mammals produce two types of melanin pigments, the black to brown eumelanin and the yellow to reddish pheomelanin [6–9].

In contrast to other biopolymers such as proteins or nucleic acids, melanin is characterized by the presence of nonhydrolyzable carbon-carbon bonds linking its monomers. This fact makes difficult to elucidate melanin structure due to the lack of effective analytical methods [10, 11].

As yet, the mechanisms regulating the pathogenesis of *melanoma malignum* have not been fully elucidated. It has not been established which exogenous (e.g., UV) or endogenous (genetic) factors play a key role in the melanoma etiopathogenesis. However, it is well known that melanoma is a highly metabolically active tumor that produces numerous substances recognized as neoplastic markers (e.g., cytokines, growth factors, apoptotic factors) used in laboratory diagnostics. The markers used for the detection of melanoma cells included some antigenic proteins as well as precursors and metabolites of melanin (assayed in serum and urine) [12].

The synthesis and accumulation of the melanins in the malignant melanoma cells are essential factors which determinate the efficacy of therapy. There are different opinions about the role of melanins in *melanoma malignum *etiopathogenesis. On the one hand, the presence of melanins can cause the negative effects in malignant cells. Firstly, melanins can bind some cancer medicines (including cytostatic drugs) and, in this way, reduce the efficiency of chemotherapy. Secondly, the excess of melanins can lead to hypoxia and reduction of the efficiency of radiotherapy. Moreover, indirect products of melanogenesis and the products arising as a result of melanins degradation are all cytotoxic. The cytotoxicity, instead of inhibiting the development of malignant cells, can turn against the immune system cells [4].

On the other hand, melanogenesis is the marker of melanocytes' differentiation, which is the opposite process to carcinogenesis [13]. One of the chemical compounds, which can suppress the proliferation and induce the differentiation of malignant cells *in vitro*, is dimethyl sulfoxide (DMSO). This highly polar organic liquid is often used as a chemical solvent. It also shows a range of pharmacological activity, like anti-inflammatory properties. DMSO induces melanogenesis and differentiation of HO melanoma cell line. However, it does not induce the dendrite-like structures in malignant cells [14].

In the present study, we developed an experimental model useful for investigations of factors controlling the processes of differentiation and melanogenesis in human melanoma cells. DMSO was used as a model compound stimulating differentiation and melanin synthesis in human A375 cell line.

The aim of the study was to characterize the chemical structure of melanin isolated from DMSO-treated human melanoma cells (A-375), using pyrolysis-gas chromatography/mass spectrometry (Py-GC/MS). In the future, structure elucidation of melanin pigments isolated from *melanoma malignum* may be helpful in the establishment of criteria for prediction of the risk of melanoma skin cancer.

## 2. Experimental

### 2.1. Materials

#### 2.1.1. Tumor Cells

The human malignant melanoma cell line A-375 was purchased from LGC Promochem (Lomianki, Poland). Malignant cell line was grown in the medium containing the following composition: 90% Minimum Essential Medium Eagle (MEM, Sigma-Aldrich), 10% fetal bovine serum (FBS, PAA), 100 U/mL penicillin, 100 *μ*g/mL streptomycin (Sigma-Aldrich), and 10 mM HEPES (Sigma-Aldrich). The cell culture was cultivated in the standard conditions: temperature was 37°C, and atmosphere was containing 95% air and 5% CO_2_. To study the cell proliferation, melanocytes were plated at an initial density of 10^3^ cells per well in 200 *μ*L of culture medium in 96-well plates. Cells were allowed to attach and grow for 24 h prior to exposure to test reagents. Cells were incubated with DMSO for 72 h.

#### 2.1.2. Melanin Isolation from A-375 Melanoma Cells

1 g of melanoma cells (A-375 cell line) was mixed with 5 mL of 1% Triton X-100 (Sigma) and incubated for 1 h at room temperature [15]. Next the sample was centrifuged (10000 ×g, 15 min), and the cell pellet was washed with phosphate buffer and once again centrifuged. The pellet was mixed with 5 mL of (5 mg/mL) sodium dodecyl sulfate (SDS) in Tris-HCl buffer (50 mM, pH = 7.4) with proteinase K (Sigma) to the final solution of 0.33 mg/mL. The mixture was incubated for 3 h in 37°C. After centrifugation (10000 ×g, 15 min), the melanin pigment was successively washed with 0.9% NaCl, methanol, and hexane and every time centrifuged (10000 ×g, 15 min). The melanin was dried at 37°C and stored in glass desiccator over P_2_O_5_.

#### 2.1.3. Synthesis of Melanin from Tyrosine Catalyzed by Tyrosinase (Tyr-Melanin)

0.004 g tyrosine hydrochloride (Sigma) was dissolved in 50 mL sodium phosphate buffer (pH 6.8), then tyrosinase 100 U/mL (Sigma, 5370 U/mg) was added, and the reaction mixtures were incubated for 48 h at 37°C with vigorous stirring and protection from light. The obtained Tyr-melanin pigment formed was collected by centrifugation (5000 ×g, 10 min) and washed several times with deionized water. To remove possible traces of tyrosinase, Tyr-melanin standard was treated with SDS and methanol, NaCl, then rewashed with deionized water, and dried to a constant weight at 37°C.

### 2.2. Methods

#### 2.2.1. Tyrosinase Activity Assay

The tyrosinase activity was assayed by the method of Slominski et al. [16]. A-375 cells were seeded in 100 mm culture dishes at a density of 1 × 10^6^ cells/dish in 12 mL of above-mentioned medium. The cells were allowed to attach and grow for 24 h. Subsequently, the culture medium was changed and the cells were treated with 0.3% and 1% DMSO for 3 and 7 days. At the end of the incubation periods, cells were washed with PBS and collected by trypsinization. Detached cells were centrifuged at 4000 g for 5 min. Subsequently, the cell pellet was lysed in 1% Triton X-100 (Sigma) in 0.1 M phosphate buffer (pH 6.8) for 30 min. Lysate was incubated with an equal volume of DOPA (3 mg/mL in 0.1 M phosphate buffer pH 6.8) for 3 h at 37°C and the absorbance was measured at 490 nm (spectrophotometer Hewlett Packard 8452A). The stimulation of tyrosinase activity following the DMSO treatment was estimated as fold increase in treated cells with respect to vehicle-treated control cells.

#### 2.2.2. Tyrosinase Expression (RT-PCR)

A-375 cells were seeded and treated as it was described previously. The transcriptional activity of tyrosinase gene was determined using a real-time RT-PCR technique. Total RNA was extracted from A-375 cells treated with DMSO for 3 and 7 days. RNA was extracted with NucleoSpin RNA II Kit (Macherey-Nagel) according to the manufacturer's instructions.

All RNA samples were treated with DNase I (Macherey-Nagel). The RNA concentration was determined using Quant-iT RiboGreen RNA Assay Kit (Invitrogen) according to the manufacturer's instructions. The primers for PCR amplification of tyrosinase transcript were designed using Primer Express 2.0 software on a sequence attained from GenBank (ref. no U01873). Primer sequences were as follows: TF 5′-CTTCGATTTGAGTGCCCCAGA-3′ and TR 5′-CCAAGCAGTGCATCCATTGAC-3′. Glyceraldehyde-3-phosphate dehydrogenase (GAPDH) RNA was used as an endogenous control. GAPDH primer pair was as follows: GF 5′-GAAGGTGAAGGTCGGAGTC-3′ and GR 5′-GAAGATGGTGATGGGATTTC-3′ [17].

The reverse transcription and amplification reactions were performed by use of the Power SYBR Green RNA-to-CT 1-Step Kit (Invitrogen). The reaction was performed in a volume of 20 *μ*L, containing 50 ng of RNA and 0,2 *μ*M primers. The cycle parameters were as follows: 30 min at 48°C, 10 min at 95°C followed by 40 cycles of 15 s at 95°C, 30 s at 54°C, and 30 s at 72°C. Under these reaction conditions, the amplification efficiencies were between 95 and 100%. The specificity of PCR reaction was confirmed by melting curve analysis and by the use of electrophoresis on 2% agarose gels, stained with ethidium bromide. The threshold cycle (Ct) values were used to determine the relative expression ratios between the controlled and treated cells. The relative expression calculations and statistical analyses were performed using the REST 2009 software [18]. Real-time RT-PCR was run in triplicate for both genes in each sample.

#### 2.2.3. Py-GC/MS of Melanin

Melanin samples were placed on the tips of ferromagnetic wires and inserted immediately into a Curie Point pyrolyser (type 795050, Pye-Unicam), coupled directly to a gas chromatograph (5890 Series II, Hewlett-Packard), and interfaced with a mass spectrometer (5989A, Hewlett-Packard). The pyrolysis (thermolysis) was carried out at 770°C for 8 s. The pyrolysis cell was kept at 220°C. GC separations of the products formed were performed on Rtx-5MS (Restek) fused-silica capillary column (5% diphenyl, 95% dimethyl polysiloxane, 60 m × 0.32 mm i.d., 0.5 *μ*m film thickness). The GC column outlet was connected directly to the ion source of a mass spectrometer. The GC/MS interface was kept at 250°C. Helium was used as the carrier gas at a flow rate of 1.8 mL/min. The GC oven temperature was programmed from 40°C (isothermal for 5 min) to 220°C at a rate of 10°C/min; the final temperature was held for 15 min. The MS conditions were as follows: electron energy, 70 eV; ion source temperature, 200°C; and quadrupole analyzer temperature, 100°C. All the mass spectra were recorded at m/z 25–200 (0–5 min) and m/z 33–500 (above 5 min). A Hewlett-Packard, ChemStation G1034C version C.02.00 software was used for data collection and mass spectra processing. The mass spectra of the thermolysis products obtained were compared with standard spectra of the Wiley Registry of Mass Spectral Data 8th Edition.

## 3. Results and Discussion

It is now well known that changes in the histone acetylation levels are involved in melanoma pathogenesis. Especially, histone hypoacetylation influences the transcriptional activity of numerous genes controlling cell cycle progression, cell signaling, differentiation, DNA repair, apoptosis, invasion, and immune response. The acetylation reaction is catalyzed by the histone acetyltransferase (HAT) and the opposite process—deacetylation—is catalyzed by histone deacetylases (HDACs). Histone deacetylase inhibitors (HDACis) inhibit cell proliferation and induce apoptosis in numerous neoplastic cell lines. They are considered as a class of new promising antineoplastic drugs, possessing chemopreventive potential. So far, clinical studies were performed with the some HDACis, for example, valproic acid, FR901228, MS-275, and SAHA [19]. DMSO is also HDAC inhibitor and hence it influences on proliferation and differentiation of various cancer cell lines.

Dimethyl sulfoxide is one of the factors, which can induce the melanogenesis. DMSO and the other factor, 6,7-dimethoxycoumarin, can induce melanogenesis even without increased tyrosine concentration in the culture medium [20, 21]. Similar effects were obtained in the experiment performed by Huberman and coworkers, where human malignant melanoma cell line HO was exposed to DMSO at concentrations of 0.5%–2%. The authors have also shown that DMSO at concentrations of 1.5% and 2% significantly inhibited proliferation of malignant cells [13]. It has been confirmed during our preliminary experiments that DMSO at concentrations of 3% and 10% has significantly decreased the proliferation of A-375 cell line and exerted strong cytotoxic effect (data not shown). We have also found that DMSO in concentrations of 0.3% and 1% did not significantly influence on the A375 cells growth.

It has been proved that DMSO can induce the differentiation of pineal cells of 8-day embryonic quail. Those cells have ability to transform into skeletal muscle fibers, pigmented epithelial cells, lens cells, and neurons. Melanogenesis is one of the earliest markers of multipotential pineal cells differentiation. In 8-day pineal, three levels of differentiation are observed. The first level includes melanin and tyrosinase; the second owns tyrosinase without pigment. Third level is tyrosinase-negative. DMSO transforms pineal cells into pigment cells including all 3 levels [22].

**Figure 1:**
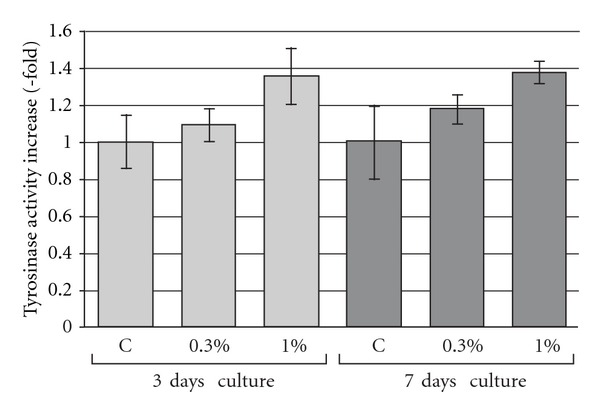
The effect of 0.3% and 1% DMSO on tyrosinase activity in A-375 melanoma cells cultured by 3 or 7 days. Each bar represents the mean ± SD; C: control.

In this study, melanogenesis was evaluated by determination of tyrosinase activity, the key enzyme of this process [23, 24]. The tyrosinase catalyzes the hydroxylation of L-tyrosine to 3,4-dihydroxyphenylalanine (L-DOPA) followed by the oxidation of L-DOPA to dopaquinone [6, 7]. Our results indicated that after 3 days of incubation of cells with DMSO at the concentrations of 0.3% and 1%, the tyrosinase activity was increased 1.09- and 1.35-fold over control, respectively, whereas after the 7 days of incubation the activity of this enzyme increased 1.17- and 1.37-fold for 0.3% and 1% DMSO treated cells, correspondingly (Figure 1), leading to an augmentation of melanogenesis process. Melanin accumulation in cells treated with DMSO was previously demonstrated by Huberman et al. and Siracký et al. [14, 20].

**Figure 2:**
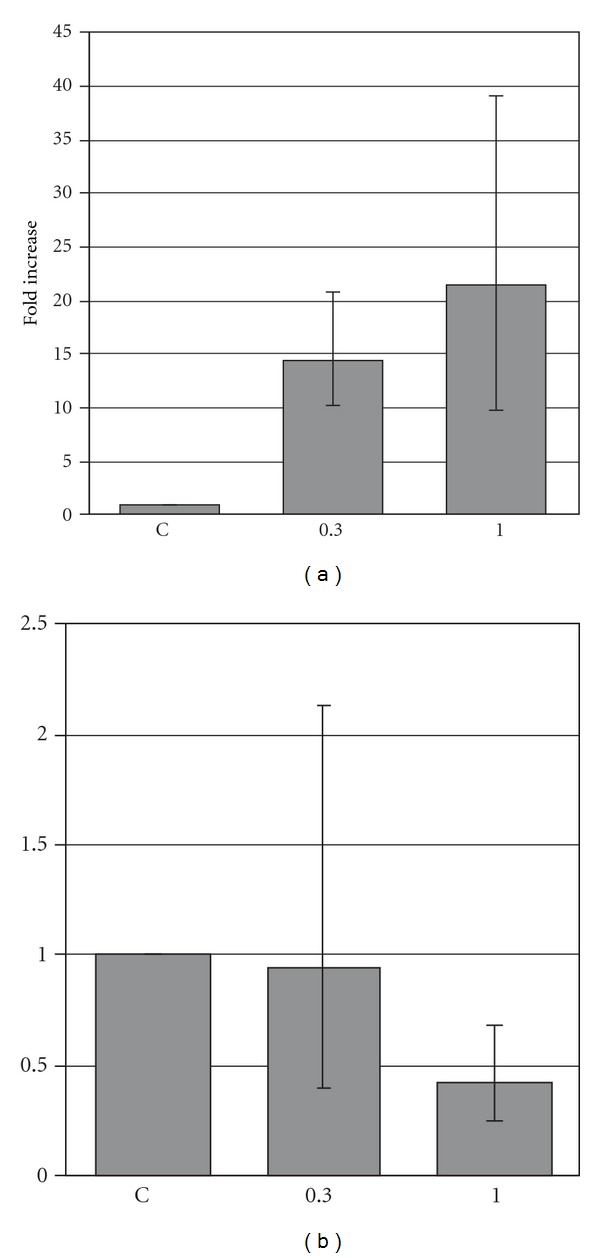
The effect of 0.3% and 1% DMSO on the transcriptional activity of tyrosinase gene in A-375 cells cultured by 3 (a) or 7 (b) days. Each bar represents the mean ± SE; Control value was taken as 1; *P < 0.05.

The tyrosinase transcript levels in A-375 cells were assessed using real-time PCR technique. Treatment of cells with 0.3% and 1% DMSO for 3 days resulted in a large increase of transcriptional activity of the tyrosinase gene (Figure 2(a)). However, after the extension of the incubation period to 7 days, levels of tyrosinase mRNA dropped below the control value (Figure 2(b)). This indicates that the augmentation of transcriptional activity of tyrosinase gene after DMSO treatment has a temporary character. It is followed by a reduction of the tyrosinase gene expression, presumably as a result of the negative feedback regulation. These results are in concordance with findings of Riley [4], who stated that increased melanogenesis with over-expression of tyrosinase in malignant melanoma was associated with defective melanosomes.

Although we observed only moderate increases of tyrosinase activity after treatment of A-375 cells with DMSO, a considerable accumulation of melanin took place in the treated melanocytes. We were not able to isolate melanin from untreated cells, whereas isolation efficiency of 4.9 mg/g was achieved after treatment of cells with 0.3% DMSO for 7 days. Similar response of that cell line was observed by Alesiani et al. [25] after exposition to 5,7-dimethoxycoumarin. The authors found fivefold increase in the melanin content within the cells after treatment with 500 *μ*M 5,7-dimethoxycoumarin, whereas tyrosinase activity was elevated 1.23-fold.

The melanin isolated from A-375 cells treated with 0.3% DMSO has been analyzed by use of Py-GC/MS technique. It can be noticed that the main pyrolysis product was 1,2-benzenediol. The other detected products were: benzene, pyrrole, pyridine, phenol, indole, and their methyl derivatives. These compounds are considered to be the most characteristic products of thermal degradation of eumelanin [26, 27].

Stępień et al. [28] have shown that the pyrolytic profile of melanin isolated from human epidermal melanocytes contained mainly compounds characteristic of eumelanins; however, the predominant product turned out to be styrene. The differences concerned the presence of traces of thiazole and methylthiazole (pyrolysis products of pheomelanin) in the pyrolysates of melanin from epidermal melanocytes.

**Figure 3:**
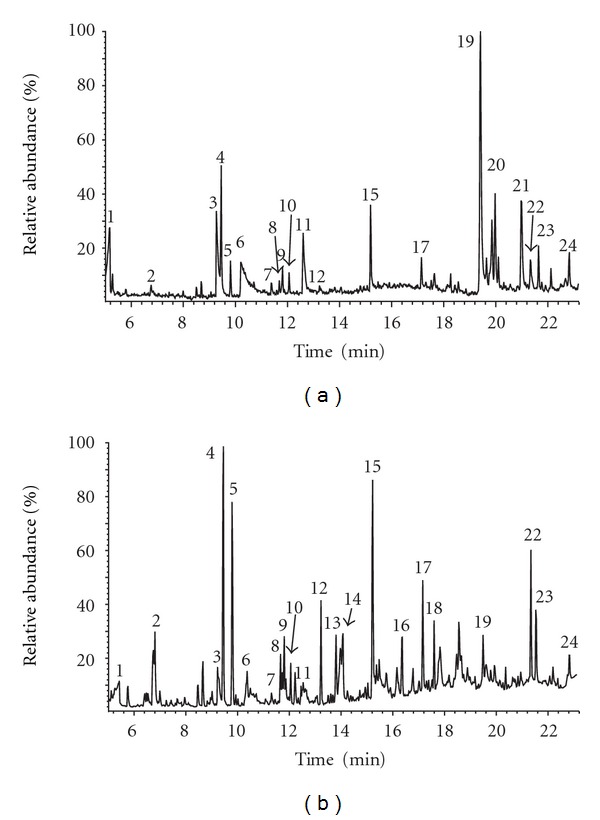
Chromatogram of the products formed during pyrolysis of (a) melanin isolated from the human melanoma malignum (A-375 cell line) treated by DMSO, and (b) model synthetic eumelanin (Tyr-melanin). Peak designation: (1) acetic acid, (2) benzene, (3) pyridine, (4) pyrrole, (5) toluene, (6) acetamide, (7) pyridine, 2-methyl-, (8) furfural, (9) pyrrole, 2-methyl-, (10) pyrrole, 3-methyl-, (11) pyridine, 4-methyl-, (12) styrene, (13) furanone, (14) cyclohexanone, (15) phenol, (16) n.i., (17) phenol, 2-methyl-, (18) phenol, 4-methyl-, (19) 1,2-benzenediol, (20) n.i., (21) 1,2-benzenediol, 4-methyl-, (22) indole, (23) 2-methoxy-4-vinylphenol, (24) indole, 3-methyl-.

We have not detected any marker products of thermal degradation of pheomelanin in the pyrolysate of natural melanin from A-375 cell line [29, 30]. Therefore, synthetic melanin obtained as a result of enzymatic tyrosine oxidation (Tyr-melanin) has been used as a standard of human eumelanin pigment. Py-GC/MS analysis revealed that pyrolytic profile of melanin derived from A-375 cells treated with DMSO (Figure 3(a)) was qualitatively similar to melanin formed from tyrosine in the presence of tyrosinase (Figure 3(b)). However, there were some differences in the quantitative relationships between particular products of thermal degradation of these melanins. The predominant pyrolysis product of Tyr-melanin was pyrrole, whereas pyrolysates of natural melanin were dominated by 1,2-benzenediol. Relatively big amounts of phenol, toluene, and indole were detected in pyrolysates of Tyr-melanin. It is possible that the lower content of compounds (pyrrole, indole, and benzene derivatives) in melanin isolated from neoplastic A-375 cells can be helpful in discrimination of melanins from *melanoma malignum*.

The melanoma cells are metabolically and produce a lot of substances recognized as neoplastic markers (some proteins, cytokines, melanin metabolites) that are used in laboratory diagnostics [12, 31]. Rosso et al. [32] have reported that pyrrole-2,3,5-tricarboxylic acid (PTCA), characteristic product of eumelanin degradation, can be a reliable marker of melanoma. 4-amino-3-hydroxy-phenylalanine (4-AHP) and 3-amino-4-hydroxyphenylalanine (3-AHP), typical products of pheomelanin degradation, could play the same role [33].

The obtained results indicate the lack of relationships between the composition of products obtained as a result of thermal degradation of melanin from A-375 cell line and melanin markers assessed in the urine of patients suffering from melanoma [33].

The elucidation of structure of melanin isolated from *melanoma malignum* may be helpful to develop criteria allowing a prediction of the risk of skin tumor.

## 4. Conclusions

Malignant melanoma is one of the most malicious tumors. The average survival time of patients with this stage of melanoma usually does not exceed 1 year. Chemotherapy or immunotherapy lengthens the survival time only to about 5 years and only in a small group of patients. In this case, there is a need to look for the new therapeutic and diagnostic solutions. Melanogenesis is a marker of the melanocyte differentiation, which is an opposite process to the carcinogenesis. Therefore, there is a possibility to evaluate the stage of malignancy by determination of the intensity of melanogenesis in malignant melanoma cells. In our study we proved that DMSO induces melanogenesis in A-375 cell line, thereby it stimulates the differentiation of melanoma cells and increases the activity of tyrosinase enzyme. However, this compound does not induce the formation of dendritic-like cells (e.g., 5,7-dimethoxycoumarin). We also proved that the predominant melanin isolated from melanoma cells is eumelanin. The results presented in our work show that DMSO is able to induce the differentiation of human melanoma cells *in vitro*. Thereby, DMSO can be used as a research model of other HDACis, which have application in the treatment of malignant melanoma.
